# A Case of Acute Hyperkalaemia of Unknown Origin During General Anaesthesia in a Rottweiler

**DOI:** 10.3390/ani15121730

**Published:** 2025-06-11

**Authors:** Dany Elzahaby, Olivier Louis Levionnois, Francesca Tosi, Ute Morath-Huss

**Affiliations:** Vetsuisse Faculty, Department of Clinical Veterinary Medicine, Section of Anaesthesiology and Pain Therapy, University of Bern, 3012 Bern, Switzerland; olivier.levionnois@unibe.ch (O.L.L.); francescatosi98@gmail.com (F.T.); ute.morath@unibe.ch (U.M.-H.)

**Keywords:** anaesthesia, hyperkalaemia, dexmedetomidine, propofol, rottweiler, complications

## Abstract

A 4-year-old neutered female rottweiler underwent three separate anaesthetic events for the diagnosis and treatment of cranial cruciate ligament rupture. The patient was otherwise healthy, with consistently normal pre-anaesthetic electrolyte values. However, during the second anaesthetic procedure, the patient developed severe hyperkalaemia with life-threatening cardiovascular compromise. Prompt treatment led to rapid normalisation of plasma potassium levels and resolution of cardiac abnormalities. This case highlights the need for further research to elucidate the mechanisms underlying this rare but serious complication. Clinicians should remain vigilant, as intra-anaesthetic hyperkalaemia, though uncommon, can be fatal. While the condition has been historically associated with greyhounds, recently reported cases in rottweilers may warrant similar concern.

## Simple Summary

Rare and unexpected complications during anaesthesia can be life-threatening if not promptly recognised and managed. This case describes a rottweiler that developed severe hyperkalaemia (elevated plasma potassium levels) during anaesthesia. Although most reports in dogs have involved greyhounds, this case adds to a small but growing number involving rottweilers. Given the risk of rapid cardiovascular compromise, clinicians should be aware of this potential complication and prepared to initiate immediate treatment to prevent a fatal outcome. Continuous monitoring, early recognition and prompt intervention are essential to ensuring patient safety during anaesthesia.

## 1. Introduction

Hyperkalaemia, though uncommon, is a life-threatening electrolyte disturbance that can occur during the peri-anaesthetic period. Its occurrence presents a significant challenge to clinicians, as prompt diagnosis and intervention are crucial for ensuring positive patient outcomes [1]. This condition can result from various causes, such as decreased renal excretion (e.g., acute renal failure or uroabdomen), acidosis (whether metabolic or respiratory), or iatrogenic factors (overzealous fluid administration or supplementation) [2,3]. Additionally, a relatively new and poorly understood phenomenon termed ‘acute unknown origin hyperkalaemia’ has been described in dogs under general anaesthesia [3]. Though the nomenclature may vary, this condition has been documented in a limited number of cases in the literature [3,4,5,6,7,8,9,10]. Potential risk factors have been linked to general anaesthesia, including the use of α_2_-adrenergic agents, such as dexmedetomidine and the phenol derivative, propofol [3,4,6].

This case report discusses a rottweiler that underwent three separate anaesthetic procedures, the first two involving dexmedetomidine and propofol and the third without these agents, only developing acute unknown origin hyperkalaemia during the second. The report aims to add to the sparse but expanding literature on this rare condition in dogs, whilst emphasising the unique aspects of this case.

## 2. Case Description

A 4-year-old neutered female rottweiler was admitted to a veterinary referral hospital in Switzerland for evaluation and management of right hindlimb lameness. Haematology and biochemistry performed on admission were unremarkable, though electrolytes were not assessed. General anaesthesia was performed for radiography. The patient received intramuscular dexmedetomidine 0.015 mg/kg (0.5 mg/mL, Orion Pharma, Switzerland) and methadone 0.2 mg/kg (10 mg/mL, Streuli Pharma AG, Uznach, Switzerland), followed by intravenous propofol 1.5 mg/kg (10 mg/mL, Fresenius Kabi, Geneva, Switzerland). The trachea was intubated, and anaesthesia was maintained with isoflurane in an oxygen/air mixture for 40 minutes. No anaesthetic complications occurred. The patient was diagnosed with a right hindlimb cruciate ligament rupture, and arthroscopy with tibial-plateau-levelling-osteotomy (TPLO) was planned.

Fifteen days later the patient re-presented for the scheduled surgical procedure. Persistent hindlimb lameness was noted, although the preanaesthetic examination was otherwise unremarkable. Premedication was administered intramuscularly, consisting of dexmedetomidine 0.01 mg/kg (0.5 mg/mL, Orion Pharma, Zug, Switzerland), methadone 0.3 mg/kg (10 mg/mL, Streuli Pharma AG, Switzerland), and acepromazine 0.03 mg/kg (10 mg/mL, Arovet AG, Dietikon, Switzerland). Once adequate sedation was achieved, an intravenous catheter was placed for drug and fluid administration. Blood was collected for haematology, biochemistry, and electrolyte analysis, which were all within normal limits except for a hyperglycaemia (12.95 mmol/L). The patient was pre-oxygenated with 4 L/min of oxygen via facemask and anaesthesia was induced with intravenous propofol 1.1 mg/kg (10 mg/mL, Fresenius Kabi, Switzerland) and ketamine 1 mg/kg (100 mg/mL, Streuli Pharma AG, Switzerland). Endotracheal intubation was performed, and anaesthesia was maintained with isoflurane as clinically required. Continuous monitoring included heart rate, respiratory rate, non-invasive blood pressure, pulse-oximetry, electrocardiography (ECG), and end-tidal CO_2_ including anaesthetic gases. Preoperative antibiotics were administered after placement of monitoring equipment with intravenous ampicillin 30 mg/kg (1000 mg/500 mg, Fresenius Kabi, Switzerland). Ringer’s acetate (Fresenius Kabi, Switzerland) was delivered at 5 mL/kg/h via an infusion pump. The right hindlimb was clipped and aseptically prepared. Under ultrasound guidance and nerve electrostimulation, femoral and sciatic nerve blocks were performed as per Campoy et al. [11], using 3.7 mL (0.5 mg/kg) of 0.5% ropivacaine (Sintetica SA, Mendrisio, Switzerland) at each site. The patient was then transferred from the anaesthesia preparation room to the operating theatre.

Toward the completion of arthroscopy and after 80 min of uneventful anaesthesia (patient parameters are provided in Supplementary Table S1), the patient developed marked bradycardia (from 81 to approximately 25 bpm) accompanied by a sudden decrease in respiratory rate. This prompted the initiation of mechanical volume-controlled ventilation at 10 mL/kg and 10 breaths per minute. Over the next 10 min, the patient developed progressively abnormal cardiac rhythms, including intermittent cardiac standstills of very short durations, alternating with intermittent phases of third-degree atrio-ventricular block. Hypotension ensued, with a mean arterial pressure of 50 mmHg. Over the following 10 min, glycopyrrolate 0.01 mg/kg IV (0.2 mg/mL, Esteve GmbH, Berlin, Germany) was administered twice with no response. Atropine 0.01 mg/kg IV (1 mg/mL, Sintetica SA, Mendrisio, Switzerland) was then administered and a blood sample collected for electrolyte analysis. The surgical team was instructed to conclude the procedure, and isoflurane was discontinued. Atipamezole 0.1 mg/kg (5 mg/mL, Orion Pharma, Switzerland) was administered intravenously to antagonise dexmedetomidine. At this time, electrolyte analysis revealed a plasma potassium concentration of 8.09 mmol/L (Table 1).

Correction of hyperkalaemia consisted of rapid acting insulin 0.1 IU/kg slowly IV (100 IU/mL, Novo Nordisk Pharma AG, Zürich, Switzerland), glucose 0.5 g/kg slowly IV (50%, B. Braun Medical AG, Melsungen, Germany), terbutaline 0.01 mg/kg SC (0.5 mg/mL, AstraZeneca GmbH, Hamburg, Germany). Additionally, calcium gluconate 50 mg/kg IV over 10 min (10%, B. Braun Medical AG, Sempach, Switzerland) was administered for its cardioprotective properties. Following treatment and the cessation or reversal of sedative/anaesthetic drugs, the patient’s heart rate rapidly increased to 120 bpm, with restoration of sinus rhythm, normotension, and a decrease in plasma potassium concentration to 4.75 mmol/L.

**Figure 1:**
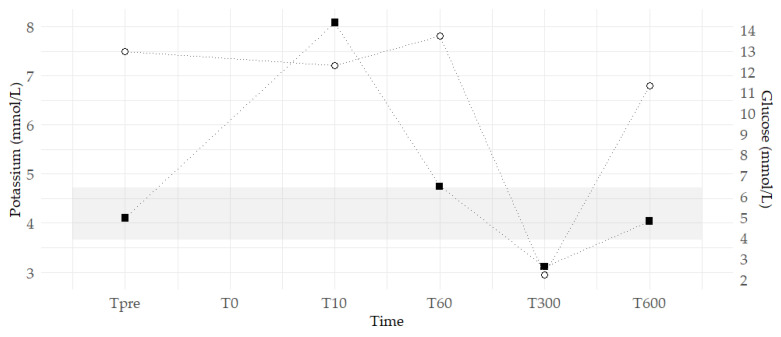
Plasma potassium and glucose concentrations during the second anaesthetic event. Open circles represent measured glucose concentrations; black squares represent measured potassium concentrations. Tpre represents the pre-induction baseline, while T0 marks the onset of observed cardiovascular abnormalities. Subsequent time points—T10, T60, T300, and T600—indicate measurements taken 10, 60, 300, and 600 min after T0, respectively. The shaded region denotes the reference interval for plasma potassium (3.66–4.72 mmol/L).

Twenty-five minutes after initiation of treatment and cessation of anaesthetic drugs, the patient awakened slowly and was transferred to the ICU for continued ECG monitoring and serial electrolyte assessments (Figure 1). Postoperative analgesia was provided with methadone 0.2 mg/kg IV, and the patient received Ringer’s acetate with a 5% glucose infusion until monitored parameters were adequately stabilised. Hypoadrenocorticism was ruled out following normal endogenous cortisol and adrenocorticotropic hormone (ACTH) stimulation test results. Echocardiography was performed to exclude cardiac disease and revealed no abnormalities. The patient was discharged after 48 h of monitoring, having consistently shown normal ECG and electrolyte parameters, including potassium, and returning to normal activity.

As the TPLO was not completed initially, the procedure was rescheduled for one month later. The patient was admitted to the hospital the night before surgery and received trazodone 8 mg/kg (100 mgs, OM Pharma, Geneva, Switzerland) and gabapentin 24 mg/kg (300 mgs, Mepha Pharma AG, Basel, Switzerland) orally both the night before and two hours prior to the procedure. Repeated haematology, biochemistry and electrolyte analyses were unremarkable, with a plasma potassium of 4.3 mmol/L. An intravenous catheter was placed, and the patient was premedicated with acepromazine (0.01 mg/kg IV) and methadone (0.3 mg/kg IV). Preoxygenation was performed using a facemask with 4 L/min of 100% oxygen. Continuous monitoring, including heart rate, respiratory rate, non-invasive blood pressure, pulse-oximetry and ECG, was initiated before induction. Anaesthesia was induced with alfaxalone 0.25 mg/kg IV (10 mg/mL, Graeub AG, Bern, Switzerland) and ketamine 2 mg/kg IV (100 mg/mL, Streuli AG, Birmensdorf, Switzerland), followed by endotracheal intubation. Anaesthesia was maintained with isoflurane, and locoregional anaesthesia was performed identically to the previous procedure. A catheter was inserted into the left dorsal pedal artery to facilitate blood sampling and continuous invasive blood pressure monitoring. Ampicillin 30 mg/kg IV was administered preoperatively.

Plasma potassium and glucose concentrations were measured every 30 min during anaesthesia and 10 min post-anaesthesia, all remaining within normal limits. Mild hypotension occurred twice and was successfully treated once with ephedrine 0.1 mg/kg IV (5 mg/mL, Bichsel AG, Bern, Switzerland) and in the other incident with a Ringer’s acetate bolus (10 mL/kg over 10 min). The procedure, including recovery, was otherwise uneventful. Postoperatively, the patient received methadone 0.1 mg/kg IV and meloxicam 0.2 mg/kg IV (Inflacam 5 mg/mL, Virbac, Zürich, Switzerland) for analgesia before being transitioned to buprenorphine 0.015 mg/kg IV (Bupaq P 0.3 mg/mL, Streuli Pharma AG, Switzerland), with pain assessed using the Glasgow Composite Measure Pain Score-Short Form (CMPS-SF) [12]. For ease of comparison, anaesthetic protocols, duration, and electrolyte parameters are summarised in Table 2.

## 3. Discussion

This case report describes a rare and life-threatening episode of acute hyperkalaemia of unknown origin during general anaesthesia in a rottweiler. The patient, a healthy rottweiler with isolated cranial cruciate ligament disease, presented with normal preanaesthetic haematology, biochemistry, and electrolyte levels, and had no history of preoperative fluid administration or potassium supplementation. Hypoadrenocorticism, as a cause of hyperkalaemia, was suspected and subsequently ruled out based on normal postanaesthetic endogenous cortisol levels and an unremarkable ACTH stimulation test. Additionally, the patient’s clinical signs completely resolved following the termination of anaesthesia and targeted potassium management. In the absence of identifiable causes and considering the timing of the hyperkalaemia, a diagnosis of anaesthesia-associated acute hyperkalaemia of unknown origin was made.

In evaluating other biochemical abnormalities observed during the peri-anaesthetic period, the patient was noted to be hyperglycaemic prior to induction (Figure 1)—a recognised effect of dexmedetomidine in dogs, primarily due to inhibition of insulin release [13]. Nonetheless, the measured glucose concentration (12.95 mmol/L) exceeded values previously reported in the literature (8.9 mmol/L *±* 1.8) [13], suggesting additional contributing factors, such as stress-induced hyperglycaemia [14]. Although acute hyperglycaemia can theoretically promote extracellular potassium shifts through osmotic redistribution, clinically significant hyperkalaemia in this context remains limited to anecdotal reports in humans [15], and is not expected in healthy individuals without underlying neuroendocrine or renal disorders [16]. This relationship remains poorly characterised in veterinary patients. Given the relative accessibility and cost-effectiveness of point-of-care glucose testing compared to electrolyte panels, further investigation into the predictive value of peri-anaesthetic hyperglycaemia for electrolyte disturbances may be warranted. Current guidelines provide no specific recommendations for the routine monitoring or management of transient hyperglycaemia in healthy animals undergoing elective procedures.

In this case, the bradycardia was unresponsive to anticholinergic treatment. Although the administered dose of atropine (0.01 mg/kg) is standard in our setting, it may have been insufficient, as higher doses are occasionally required to achieve a therapeutic effect [17]. However, prior administration of glycopyrrolate also failed to resolve the bradycardia, further supporting the hypothesis that the bradycardia was attributable to an electrolyte imbalance rather than increased vagal tone [18]. Notably, a retrospective study investigating anaesthesia-associated acute hyperkalaemia, identified bradycardia that was minimally responsive to anticholinergic agents as a primary clinical warning sign [3].

Hyperkalaemia was managed using multiple interventions, primarily aimed at shifting potassium from the extracellular to the intracellular compartment. Insulin facilitates this shift by up-regulating the Na^+^/K^+^-ATPase pump in skeletal muscle [19]. Consequently, insulin administration also lowers plasma glucose concentrations, as observed in this patient; hence glucose supplementation is recommended. Terbutaline, a β_2_-adrenergic agonist, was also administered; like insulin, it promotes the intracellular shift of potassium by stimulating the Na^+^/K^+^-ATPase pump [19]. Although its efficacy can be variable, it is generally considered an adjunctive therapy, with effectiveness enhanced when combined with insulin [19,20,21]. Calcium gluconate was additionally given. While it does not lower serum potassium, it stabilises cardiac membranes by increasing the threshold potential, thereby restoring the gradient between the resting and threshold membrane potentials [20]. Also, atipamezole may have concurrently contributed to the reduction in plasma potassium, as it has been associated with attenuation of medetomidine-related hyperkalaemia in multiple species [6,22,23].

Throughout anaesthesia, the patient received Ringer’s acetate, which contains approximately 4 mmol/L of potassium [24]—lower than concentrations typically seen in hyperkalaemia. Although some clinicians prefer to administer potassium-free solutions, such as normal saline, during the management of hyperkalaemia, current evidence does not support this practice [25,26]. Multiple studies in humans have shown that buffered solutions like Ringer’s acetate or lactated Ringer’s solution are associated with a lower risk of acidosis and either have no effect on, or may even reduce, serum potassium concentrations compared to normal saline [25,26].

In human medicine, ‘acute unknown origin hyperkalaemia’ or ‘intra-anaesthetic hyperkalaemia’ has been linked to propofol use [27,28]. Propofol is thought to contribute to hyperkalaemia by impairing mitochondrial fatty acid metabolism, disrupting the transcellular movement of potassium, and reducing beta-adrenoceptor responsiveness [27]. Thus, impacting intracellular potassium movement, ultimately leading to increased extracellular potassium levels. While this potential adverse reaction has also been queried in veterinary medicine [4,5], the widespread use of propofol without frequent reports of hyperkalaemia suggests that, if a causal relationship exists, it is exceptionally rare.

Similarly, α_2_-adrenergic agents such as dexmedetomidine and medetomidine have been identified as potential risk factors for intra-anaesthetic hyperkalaemia in veterinary medicine [3]. Tisotti et al. [3] reported 13 cases of acute unknown origin hyperkalaemia in dogs, all involving α_2_-adrenergic agents in combination with opioids in the premedication protocol. The proposed mechanism involves stimulation of pancreatic α_2_-receptors and subsequent interference with transcellular potassium pumps, resulting in disrupted potassium homeostasis. Although the exact pathophysiological pathway remains incompletely understood, this association stresses the need for additional research into the pharmacodynamic properties of α_2_-adrenergic agents.

Tisotti et al. [3] further proposed that prolonged maintenance anaesthesia may contribute to the development of hyperkalaemia. In the present case, the first anaesthetic event—in which dexmedetomidine and propofol were administered—was uneventful and completed within 40 minutes. In contrast, during the second procedure using the same agents, hyperkalaemia-induced bradyarrhythmias developed after 80 min of maintenance anaesthesia. This observation aligns with the findings of Tisotti et al., which reported that all dogs exhibiting clinical signs of hyperkalaemia had received maintenance anaesthesia exceeding 60 minutes. Other studies have similarly identified anaesthetic duration as a positive predictor of plasma potassium concentrations [22], suggesting that the length of anaesthesia may be an important factor in the development of clinically significant hyperkalaemia. However, in this case, hyperkalaemia did not occur during the third and longest procedure, highlighting the multifactorial nature of this complication.

Intra-anaesthetic hyperkalaemia has been repeatedly reported in greyhounds [5,8,9,10], leading to speculation of a breed predisposition. Similarly, rottweilers may also be at increased risk, as this case adds to previous reports [4,7], suggesting a possible breed-specific susceptibility. This observation prompts the need for further investigation into potential genetic factors that may increase vulnerability. Identifying such risks could help guide anaesthetic decision-making and improve patient safety in predisposed breeds.

This case stresses the importance of vigilance in managing peri-anaesthetic hyperkalaemia, particularly in patients with bradycardia that is unresponsive to anticholinergic treatment. Efforts to minimise the duration of anaesthesia may help reduce the risk of developing this condition, although this is not always clinically feasible. Clinicians should also remain alert to the possibility of hyperkalaemia in breeds where it has been repeatedly reported, most notably greyhounds, and to a lesser extent, rottweilers, particularly following administration of α_2_-adrenergic agonists. While not yet fully characterised, marked hyperglycaemia may also warrant closer monitoring of electrolyte levels. In this case, rapid identification and intervention were likely critical in preventing a fatal outcome. This case also demonstrates the value of on-site electrolyte analysis to allow timely assessment when concerns arise.

ECG monitoring is equally essential, as arrhythmias can serve as an early indicator of hyperkalaemia, enabling prompt recognition and management. Early ECG changes may include narrow, peaked T waves, while more severe hyperkalaemia can lead to QRS widening. Furthermore, hyperkalaemic arrhythmias can be highly variable, presenting as either bradyarrhythmias or tachyarrhythmias, irrespective of potassium concentrations [29]. It is important to note, however, that routine clinical monitoring may limit detailed waveform analysis in a practical setting. Nevertheless, recognising deviations from baseline remains crucial during anaesthetic monitoring.

Given these risks, anaesthetic protocols should incorporate comprehensive monitoring and preparedness for electrolyte disturbances to mitigate perioperative complications. Importantly, the 2025 American College of Veterinary Anesthesia and Analgesia monitoring guidelines list continuous ECG analysis as a minimum requirement during anaesthesia, while routine electrolyte monitoring is omitted from the recommendations—aligning with equivalent human anaesthetic guidelines [30,31]. Despite this, clinicians should recognise that intra-anaesthetic hyperkalaemia can occur in both dogs and domestic cats [23], particularly in certain breeds, and access to on-site electrolyte monitoring may be life-saving in such cases.

## 4. Conclusions

In this case, acute, life-threatening hyperkalaemia of unknown origin occurred during anaesthesia in a healthy rottweiler. Prompt recognition and targeted management were crucial to the patient’s recovery and favourable outcome. The absence of identifiable underlying disease, combined with the temporal association with anaesthetic agents, suggests an anaesthesia-related mechanism. Consistent with the literature, prolonged anaesthetic duration alongside administration of dexmedetomidine or propofol, may have contributed to the development of this condition. Nevertheless, further research is needed to better understand the pathophysiology and risk factors associated with this rare but potentially fatal complication in dogs.

## Figures and Tables

**Table 1:** Venous blood gas and electrolyte analyses before and 30 min following treatment for hyperkalaemia. Samples collected from the right cephalic vein and placed in heparinised sample tubes.

Parameter	Result (Before Treatment)	Result (After Treatment)	Reference
pH	7.14	7.14	7.35–7.45
PvCO_2_ (mmHg)	63.9	58.2	28.6–44.7
HCO_3_^−^ (mmol/L)	21.2	19.2	19.7–24.8
Base Excess (mmol/L)	−8.8	−10.5	−6.7–1.5
Na^+^ (mmol/L)	145	150	143.7–151.1
K^+^ (mmol/L)	8.09	4.75	3.66–4.72
iCa^2+^ (mmol/L)	1.36	1.48	1.23–1.4
Cl^−^ (mmol/L)	114	117	109–117
Anion Gap (mmol/L)	18.2	18.1	11.6–21.2
Glucose (mmol/L)	12.3	13.7	3.9–6.4
Lactate (mmol/L)	0.83	1.59	0.43–2.1

**Table 2:** Details surrounding the second and third anaesthetic events; these include duration of maintenance anaesthesia, serum potassium, glucose concentrations and anaesthetic protocols.

	2nd Anaesthetic Event	3rd Anaesthetic Event
Maintenance Anaesthesia (mins) ^1^	80	240
Serum Potassium (mmol/L) ^2^	3.1–8.09 ^3^ (RI: 3.66–4.72)	3.4–4.33 (RI: 3.66–4.72)
Serum Glucose (mmol/L) ^2^	2.2–12.3 ^3^ (RI: 3.9–6.4)	4.6–5.3 (RI: 3.9–6.4)
Premedication	Dexmedetomidine 0.01 mg/kg IM Acepromazine 0.03 mg/kg IM Methadone 0.3 mg/kg IM	Acepromazine 0.01 mg/kg IV Methadone 0.3 mg/kg IV
Induction	Propofol 1.1 mg/kg IV Ketamine 1 mg/kg IV	Alfaxalone 0.25 mg/kg IV Ketamine 2 mg/kg IV
Maintenance	Isoflurane	Isoflurane

^1^ Duration includes the time elapsed under isoflurane anaesthesia. ^2^ Range includes all serum concentrations measured perioperatively. ^3^ Specific values observed during the intra-anaesthetic critical event.

## Data Availability

The original contributions presented in this case are included in the article. Further inquiries can be directed to the corresponding author.

## Supplementary Materials

The following supporting information can be downloaded at: https://www.mdpi.com/article/10.3390/ani15121730/s1, Table S1: Monitored physiologic parameters preceding the critical event during the second anaesthetic.

## Abbreviations

The following abbreviations are used in this manuscript:

TPLO	Tibial-plateau-levelling-osteotomy
ECG	Electrocardiography
ACTH	Adrenocorticotropic Hormone
CMPS-SF	Composite Measure Pain Score-Short Form
