# Supplementary material for: A Case of Acute Hyperkalaemia of Unknown Origin During General Anaesthesia in a Rottweiler
Source: Animals (Basel). 2025 Jun 11;15(12):1730. doi: 10.3390/ani15121730 (PMC12189807; doi:10.3390/ani15121730)
Supplement: Supplementary file 1 [file animals-15-01730-s001.zip › animals-3610685-supplementary.pdf]

Table S1: Monitored parameters preceding the critical event, measured using non-invasive blood pressure (NIBP), end-tidal carbon dioxide ($EtCO_2$), end-tidal isoflurane (EtIso), and pulse oximetry (SpO2). Time0 represents the values recorded at the onset of induction. Subsequent time points –Time15, Time30, Time45, Time60 and Time75—correspond to parameters measured at 15, 30, 45, 60, and 75 minutes after induction, respectively. Time60 corresponds with initial incision.

	HR	NIBP (Sys/Dia (Map))	SpO_2 (%)	$EtCO_2$ (%)	EtIso (%)	Temperature (°C)
**Time0**	52	130/95 (110)	96	48	0.7	--
**Time15**	57	130/103 (112)	96	51	1.3	--
**Time30**	60	125/98 (105)	97	54	1.1	37.9
**Time45**	72	124/82 (100)	96	57	1.1	37.4
**Time60**	78	112/69 (87)	96	60	1.2	37.0
**Time75**	81	112/68 (80)	95	58	1.1	--
